# Resistance to Dolutegravir in Treatment-experienced Patients in South Africa: A Retrospective Cohort Study

**DOI:** 10.1097/QAI.0000000000003657

**Published:** 2025-03-03

**Authors:** Ying Zhao, Melanie Holtman, Vanessa Mudaly, Gert van Zyl, Gary Maartens, Graeme Meintjes

**Affiliations:** a Division of Infectious Diseases and HIV Medicine, Department of Medicine, University of Cape Town, Cape Town, South Africa; b Western Cape Government Department of Health and Wellness, Cape Town, South Africa; c Division of Medical Virology, Stellenbosch University, Stellenbosch, South Africa; d Division of Clinical Pharmacology, Department of Medicine, University of Cape Town, Cape Town, South Africa; e Wellcome Centre for Infectious Diseases Research in Africa, Institute of Infectious Disease and Molecular Medicine, University of Cape Town, Cape Town, South Africa; f Department of Medicine, University of Cape Town, Cape Town, South Africa; g Blizard Institute, Faculty of Medicine and Dentistry, Queen Mary University of London, London, United Kingdom

**Keywords:** Antiretroviral therapy, dolutegravir, HIV, virologic failure, resistance

## Abstract

**Background:**

Dolutegravir resistance has been reported more frequently in patients with prior treatment experience compared to those on dolutegravir in first-line antiretroviral therapy (ART). The widespread use of dolutegravir in resource-limited programmatic settings might facilitate the emergence of resistance. Data on the prevalence of dolutegravir resistance from programmatic settings in Africa are scarce.

**Methods:**

This retrospective observational cohort study assessed dolutegravir resistance in routine care settings of the Western Cape provincial public healthcare sector program between February 2021 and June 2024. Treatment-experienced adults who developed virologic failure (two HIV-1 RNA ≥1000 copies/mL), who had received dolutegravir-based ART for >24 months, were eligible for genotypic antiretroviral resistance testing (GART). Drug resistance mutations (DRMs) and resistance levels were classified using the Stanford database.

**Results:**

Among 99 eligible patients, 76 had GART performed, and 68 had successful sequences. Among these 68, 43 (63%) had dolutegravir DRMs with: 1 potential low, 1 low, 15 intermediate, and 26 high resistance levels. The median time on dolutegravir-based ART was 24 months (IQR, 23–31). Of the 43 patients with dolutegravir DRMs, 21 (49%) were receiving zidovudine-lamivudine-dolutegravir and 19 (44%) were receiving tenofovir-lamivudine-dolutegravir; 42/43 had prior ART experience.

**Conclusions:**

Over 60% of patients with prior treatment experience who had been on dolutegravir-based ART for over two years and experienced virologic failure had intermediate or high level dolutegravir resistance. This suggests that criteria for GART used are too stringent, which has resource implications in programmatic settings where access to resistance testing for individual management is limited.

## Introduction

As of mid-2021, 110 low- to middle-income countries (LMICs) had transitioned to generic dolutegravir and an estimated 22.2 million adults in LMICs were receiving dolutegravir-based antiretroviral therapy (ART).^1^ Large-scale transitioning to dolutegravir-based ART in resource-limited programmatic settings, where patients remain longer on failing non-nucleoside reverse transcriptase inhibitor (NNRTI)-based first-line ART and are often switched to dolutegravir-based ART without HIV-1 RNA testing, might facilitate the emergence of dolutegravir resistance given the high prevalence of nucleoside reverse transcriptase inhibitor (NRTI) resistance in people with virologic failure on NNRTI-based ART.^2^ Higher rates of dolutegravir resistance have been reported in patients with prior regimen failure and underlying NRTI resistance compared to those never having failed a regimen (i.e. have fully active NRTIs).^2,3^ As dolutegravir is rapidly rolled out, surveillance of drug resistance is crucial to determine the need for, optimal timing, and cost-effectiveness of genotypic antiretroviral resistance testing (GART) in public sector programs. Data on the prevalence and pattern of dolutegravir resistance from Africa remain scarce.

The 2023 South African National Department of Health antiretroviral guidelines recommend recycling tenofovir between first- and second-line ART, instead of switching to zidovudine, as had previously been recommended.^4^ This policy is based on evidence that maintaining tenofovir and lamivudine with dolutegravir achieved a high proportion of virologic suppression in second-line ART.^5,6^ In the Western Cape Province, in line with national guidelines, patients with prior treatment experience on dolutegravir-based ART for >24 months and having virologic failure despite adequate adherence qualified for GART to assess for dolutegravir resistance and guide selection of an alternative regimen. In August 2024, the provincial GART eligibility timeframe was adjusted to include treatment-experienced patients failing dolutegravir-based ART within 24 months of switching. We conducted a retrospective observational cohort study to describe the proportion and pattern of dolutegravir drug resistance mutations (DRMs) among patients in whom GART was approved in the Western Cape provincial public healthcare sector program under these policies.

## Methods

### Study population and eligibility criteria

In mid-2022, the estimated HIV prevalence in the Western Cape Province was 11.2% (95% confidence interval, 10.6%–11.6%) among those aged 15–49 years.^7^ Among 328943 adults on ART, 243228 (74%) were receiving dolutegravir-based regimens.^8^ Applications for GART are submitted to Service Priorities Coordination at the Provincial Department of Health and Wellness, which assesses eligibility for GART. The Provincial Third Line Committee includes HIV expert clinicians and virologists, who advise on the appropriate choice of an alternative regimen for patients who develop dolutegravir resistance after reviewing treatment history and GART results. We screened all applications between February 2021 and June 2024 and included consecutive patients with virologic failure on dolutegravir-based ART, and in whom GART was approved. Virologic failure on dolutegravir-based ART was defined as HIV-1 RNA ≥1000 copies/mL on at least two occasions. Patients were followed up at local clinics with HIV-1 RNA performed 3 months after initiation of dolutegravir-based ART. If the 3-month HIV-1 RNA was <50 copies/mL, HIV-1 RNA monitoring was recommended at 10 months and 22 months. If HIV-1 RNA was ≥50 copies/mL at any stage, patients received adherence counselling and HIV-1 RNA repeated in 3 months.^4^ Eligibility for GART were: duration of dolutegravir-based ART for >24 months, confirmed virologic failure, adherence >80% assessed by clinic visit attendance or pharmacy refills over the past 6–12 months, and being treatment-experienced with virologic failure on at least one prior regimen. The Provincial Third Line Committee approved GART for selected patient who had received dolutegravir-based ART for shorter duration (<24 months) due to clinician motivation. Reasons for earlier GART included virologic failure following a drug-drug interaction that could have substantially decreased dolutegravir concentrations and persistent viraemia despite several adherence interventions. Patients aged <18 years old were excluded.

### Procedures

Samples were collected for GART at the time of virologic failure on dolutegravir-based ART and prior to switching to an alternative regimen. GART was performed with Sanger sequencing using an assay developed by the Centers for Diseases Control and Prevention and distributed by Thermo-Fisher Scientific for *protease* and *reverse transcriptase* and an *integrase* homebrew method developed to cover diverse HIV-1 group M subtypes,^9,10^ at the National Health Laboratory Service Virology Laboratory at Tygerberg Hospital in Cape Town, South Africa. Drug-susceptibility prediction was performed with the Stanford HIV drug resistance database (HIVdb) algorithm (version 8.9–9.6).^11^ An electronic database was designed to record clinical data of patients failing second-line ART in whom a resistance test was approved from the GART application forms submitted and the electronic Provincial Single Patient Viewer system.

### Outcome measures

The primary outcome was emergence of dolutegravir DRMs in patients who were known to have developed virologic failure on dolutegravir-based ART. We defined dolutegravir DRMs as all mutations associated with integrase strand transfer inhibitors (INSTIs) by the Stanford HIVdb algorithm, including major, accessory, and other mutations.^12^ We categorised dolutegravir resistance level using the Stanford HIVdb algorithm as susceptible (score <10), potential low (10–14), low (15–29), intermediate (30–59), or high (≥60). We reviewed and summarised clinical characteristics, ART history, HIV-1 RNA levels, and CD4 cell count of patients with virologic failure on dolutegravir-based ART and in whom GART was approved. We summarised GART results and subsequent management in the ART program of patients who developed dolutegravir resistance.

### Ethical considerations

This study was approved by the Human Research Ethics Committee (HREC) of the University of Cape Town (reference 104/2022). As routine clinical data were collected in the database registry and used for observational research, which carried minimal risk to patients, a waiver for the requirement of informed consent was granted with the registry approved by the HREC at the University of Cape Town (reference R013/2021). Patient identifiers were removed from the datasets for analysis.

## Results

We reviewed 99 applications received by the Western Cape Provincial Third Line Committee and approved for integrase gene sequencing between February 2021 and June 2024. Two applications were not approved due to reported current poor adherence. Demographics and clinical characteristics at the time of application are summarised in Table 1. Of these, 76 patients had GART performed, and 68 samples were successfully sequenced (8 did not amplify integrase). Reasons for having no GART performed included re-suppression of HIV-1 RNA with enhanced adherence counselling and loss to follow-up. Reasons for failed amplification included low HIV-1 RNA levels, poor sample quality, and primer mismatch.

Among these 68 patients, 43 (63%) had dolutegravir DRMs with: 1 potential low-level, 1 low-level, 15 intermediate-level, and 26 high-level dolutegravir resistance. Clinical characteristics and details of the resistance pattern of these 43 cases are summarized in Supplementary Table S1 and Supplementary Table S2. Among these 43 patients, the median age was 38 years (interquartile range [IQR], 34–41), 58% female, and patients had a median of 24 months of experience on dolutegravir-based ART (IQR, 23–31; range, 6–60). The median HIV-1 RNA at the time of virologic failure on dolutegravir-based ART was 4.1 log_10_ copies/mL (IQR, 3.8–4.9). The median duration of viremia on dolutegravir-based ART, defined as the time from the first HIV-1 RNA >1000 copies/mL after initiating dolutegravir-based ART to the application date for GART, was 15 months (IQR, 8–21). The most frequent DRMs in the integrase region were E138K (n=20), G118R (n=19), and R263K (n=16). Of the 43 patients with dolutegravir DRMs, 19 (44%) were receiving tenofovir-lamivudine-dolutegravir (TLD), 21 (49%) were receiving zidovudine-lamivudine-dolutegravir (ALD), 2 (5%) were receiving abacavir-lamivudine-dolutegravir, and 1 (2%) was receiving ALD with ritonavir-boosted darunavir. Among the 19 on TLD, 1 patient reported being ART naïve when starting TLD, 4 had switched from NNRTI-based first-line ART, 8 from protease inhibitor-based second-line ART, and 6 from ALD as a second-line regimen. After detection of dolutegravir resistance, all patients were switched to darunavir-based ART as recommended by the Provincial Third Line Committee (Supplementary Table S1). Among those with HIV-1 RNA performed after switching to darunavir-based ART, 34/38 (89%) achieved HIV-1 RNA <400 copies/mL.

## Discussion

We observed a high proportion with dolutegravir resistance (63% of those who had successful GART) among treatment-experienced patients who developed virologic failure on dolutegravir-based ART. Many patients with resistance detected had been on dolutegravir for two years or longer due to the GART eligibility criteria. Our findings highlight the need for less stringent GART criteria to ensure resistance is detected earlier to allow appropriate treatment modification among patients with virological failure on dolutegravir-based ART in programmatic settings.

The results from our study align with the WHO updated global surveillance report on resistance to dolutegravir and other antiretroviral drugs, published in February 2024, demonstrating high prevalence of dolutegravir resistance in routine care settings among patients with viraemia on dolutegravir-based regimens.^3^ In a national cross-sectional survey conducted among 6462 adults in Malawi who were transitioned from NNRTI-based first-line ART to TLD without pre-switch HIV-1 RNA testing in 2021, patients with a repeat HIV-1 RNA >1000 copies/mL after 3 months of intensive adherence support were eligible for GART. Of 27 patients with integrase gene sequencing results, 8 (30%) harboured dolutegravir DRMs.^13^ An increasing trend of dolutegravir resistance among adults receiving dolutegravir-based ART with HIV-1 RNA ≥1000 copies/mL and confirmed dolutegravir exposure using liquid tandem chromatography mass spectrometry was observed between two South African national surveys (2.7% and 11.9% in 2021 and 2022, respectively).^14^ The higher proportion with dolutegravir resistance in our cohort may be attributed to the presence of several risk factors for dolutegravir resistance,^15–17^ including reported prior ART experience (as observed in 42 of our 43 cases), longer duration of dolutegravir-based ART (median 24 months), and persistent viraemia on dolutegravir-based ART (median 15 months of viraemia), which may lead to progressive accumulation of dolutegravir DRMs.^18^

We observed a substantial proportion of those with dolutegravir resistance (56%) were within 24 months of initiating dolutegravir-based ART. In a prospective cohort study of 1892 Malawian patients who were transitioned from NNRTI-based first-line ART to TLD, two cases of dolutegravir resistance developed by 6 months; both patients were viraemic at switch and received dolutegravir with no predicted active NRTIs.^19^ South African guidelines at the time of the study limited access for GART to patients on dolutegravir-based ART for >24 months and failing despite adequate adherence, based on cost considerations. Our findings of a high prevalence of dolutegravir resistance using these criteria as well as detection of dolutegravir resistance in some patients on dolutegravir-based ART for less than 24 months indicate that this recommendation should be reconsidered to allow earlier GART within 24 months of initiating dolutegravir-based ART. GART is rationed in resource-limited settings given the limited laboratory capacity and high costs.^20^ In most, if not all LMICs outside South Africa, access to GART is very limited and integrase gene sequencing is not routinely available for individual clinical care.^13^ Our findings suggest the need for making GART available in such settings to minimise the preventable amplification and transmission of drug-resistance HIV with mechanisms in place to ensure those at the highest risk of developing dolutegravir resistance get GART access. Further research is needed to develop cost-effective algorithms, ideally using pharmacologic metrics of ART adherence such as tenofovir diphosphate in dried blood spots or point-of-care urine-based tenofovir immunoassay,^21,22^ to identify patients at the highest risk of developing dolutegravir resistance to rationally direct resources for resistance testing.

Our study has limitations. First, the retrospective design introduces sources of bias, particularly in the selection of cases. Second, the small sample size limited our ability to assess associations with dolutegravir resistance. Third, resistance data before switching to dolutegravir-based ART were not available. Fourth, most of our patients (98%) had switched from NNRTI- or protease inhibitor-based ART to dolutegravir-based ART with virologic failure on at least one prior regimen. Our results are not generalizable to viraemic patients who started on dolutegravir-based ART and never having failed a prior regimen. Fifth, the proportion of people failing dolutegravir-based ART in the Western Cape Province was unknown. The high proportion with dolutegravir resistance we observed may be due to selection bias for resistance testing and may not be generalizable to all treatment-experienced people with virologic failure on dolutegravir-based ART.

## Conclusion

Among treatment experienced patients who were known to have developed virologic failure on dolutegravir-based ART in a South African public healthcare sector program, over 60% had intermediate-level or high-level dolutegravir resistance. To ensure the continued efficacy of TLD in programmatic settings, developing cost-effective algorithms to prioritize a subset of individuals receiving dolutegravir-based ART who experience virologic failure and are at higher risk of having developed dolutegravir resistance for integrase resistance testing is crucial.

## Supplementary Material

Supplemental Digital Content

## Figures and Tables

**Table 1:** Demographic and Clinical Characteristics

Variable	Patients, No. (%)^a^ (n = 99)
Age, median (IQR), years	39 (34-44)
Female sex	65 (65.7)
CD4 cell count, median (IQR), cells/μL^b^	159 (71-269)^c^
HIV-1 RNA, median (IQR), log_10_ copies/mL^b^	4.5 (3.9-5.1)^d^
Duration of ART exposure, median (IQR), years	8.7 (6.7-12.0)^e^
Duration of DTG exposure, median (IQR), months	24 (21-31)^e^
Duration of viraemia, median (IQR), months^f^	14 (8-23)^e^
Current ART regimen at the time of DTG regimen failure
TDF/3TC/DTG	49 (49.5)
TDF/3TC/DTG/DRV/r	3 (3.0)
TDF/3TC/DTG/DRV/r/ETR	1 (1.0)
AZT/3TC/DTG	38 (38.4)
AZT/3TC/DTG/DRV/r	2 (2.0)
ABC/3TC/DTG	6 (6.1)
Dolutegravir resistance status at the time of GART^g,h^	
Fully active	25 (36.8)
Potential low-level resistance	1 (1.5)
Low-level resistance	1 (1.5)
Intermediate-level resistance	15 (22.1)
High-level resistance	26 (38.2)
Major INSTI mutations^h,i^	
0	26 (38.2)
1–2	22 (32.3)
≥3	20 (29.4)

a Data represent number (%) unless otherwise specified.

b The data for CD4 cell count and HIV-1 RNA were recorded at the time of virologic failure on DTG-based regimen.

c Denominator: n = 87.

d Denominator: n = 94.

e Denominator: n = 96.

f From the time of the first HIV-1 RNA >1000 copies/mL on the DTG-based regimen to the application date for GART.

g Resistance was classified with the Stanford algorithm as susceptible (score <10), potential low (10–14), low (15–29), intermediate (30–59), or high (≥60).

h Denominator indicates the number of patients with available viral sequences: n = 68.

i Major INSTI mutations were defined as: T66A/I, G118R, E138A/E/K/T, G140A, S147G, Q148R, N155H, and R263K/R.

ART, antiretroviral therapy; GART, genotypic antiretroviral resistance testing; IQR, interquartile range; DTG, dolutegravir; ABC, abacavir; 3TC, lamivudine; TDF, tenofovir disoproxil; AZT, zidovudine; DRV/r, ritonavir boosted darunavir; ETR, etravirine; INSTI, integrase strand transfer inhibitor.
