# Supplementary material for: Resistance to Dolutegravir in Treatment-experienced Patients in South Africa: A Retrospective Cohort Study
Source: J Acquir Immune Defic Syndr. Author manuscript; Available in PMC 2025 Jul 1. (PMC7617472; doi:10.1097/QAI.0000000000003657)
Supplement: Supplemental Digital Content [file EMS203509-supplement-Supplemental_Digital_Content.docx]

**Supplementary Table S1. Clinical Characteristics of Patients with Dolutegravir Drug Resistance Mutations**

| **Case No., Sex, Age, years^a^** | **Current Regimen (Duration, mo)** | **Previous Regimens^b^** | **HIV-1 RNA, log_10_ copies/mL^c^** | **CD4 Cell Count, cells/μL^c^** | **Viraemia Duration, mo^d^** | **DTG Resistance Level^e^** | **Subsequent Regimen** |
| --- | --- | --- | --- | --- | --- | --- | --- |
| 1, F, 28 | AZT/3TC/DTG (14) | TDF/FTC/EFV, AZT/3TC/LPV/r | 4.79 | 429 | 3 | High | TDF/FTC/DRV/r |
| 2, M, 20 | TDF/3TC/DTG (18) | D4T/3TC/EFV, TDF/FTC/EFV | 4.89 | 42 | 18 | Intermediate | TDF/3TC/DTG/DRV/r |
| 3, M, 44 | AZT/3TC/DTG (24) | TDF/FTC/EFV | 3.68 | 234 | 12 | High | TDF/FTC/DRV/r |
| 4, M, 48 | TDF/3TC/DTG (13) | TDF/FTC/EFV, AZT/3TC/LPV/r, TDF/FTC/ATV/r | 5.26 | 94 | 8 | Intermediate | TDF/FTC/DRV/r |
| 5, F, 61 | AZT/3TC/DTG (11) | D4T/3TC/EFV, TDF/3TC/LPV/r | 3.87 | 244 | 7 | Intermediate | TDF/3TC/DTG/DRV/r |
| 6, F, 33 | AZT/3TC/DTG (19) | TDF/FTC/EFV | 3.66 | 270 | 18 | Intermediate | TDF/FTC/DRV/r |
| 7, M, 49 | AZT/3TC/DTG (60) | D4T/3TC/NVP, TDF/FTC/EFV | 4.93 | 133 | 13 | High | TDF/FTC/DRV/r |
| 8, F, 36 | AZT/3TC/DTG (24) | TDF/FTC/EFV | 3.05 | 346 | 11 | Potential low | TDF/3TC/DTG/DRV/r |
| 9, F, 40 | AZT/3TC/DTG (23) | TDF/FTC/EFV | 4.09 | 93 | 8 | High | TDF/FTC/DRV/r |
| 10, M, 33 | AZT/3TC/DTG (24) | TDF/FTC/EFV | 5.04 | 265 | 20 | High | TDF/FTC/DRV/r |
| 11, F, 40 | AZT/3TC/DTG (24) | D4T/3TC/NVP, TDF/FTC/EFV | 4.67 | 87 | 19 | High | TDF/FTC/DRV/r |
| 12, F, 34 | AZT/3TC/DTG (21) | TDF/FTC/EFV | 3.40 | 847 | 13 | High | TDF/FTC/DRV/r |
| 13, M, 41 | AZT/3TC/DTG (20) | D4T/3TC/NVP | 4.02 | ND | 17 | High | TDF/FTC/DRV/r |
| 14, M, 51 | TDF/3TC/DTG (11) | TDF/FTC/EFV, AZT/3TC/LPV/r | 5.68 | 159 | 5 | Intermediate | AZT/3TC/DRV/r |
| 15, M, 39 | TDF/3TC/DTG (23) | TDF/FTC/EFV | 3.89 | 379 | 3 | High | TDF/FTC/DRV/r |
| 16, F, 39 | TDF/3TC/DTG (28) | TDF/FTC/EFV, TDF/FTC/ATV/r | 3.68 | 261 | 2 | Intermediate | TDF/3TC/DRV/r |
| 17, F, 40 | TDF/3TC/DTG (23) | TDF/FTC/EFV | 2.93 | 380 | 1 | Intermediate | TDF/FTC/DRV/r |
| 18, F, 32 | TDF/3TC/DTG (6) | TDF/FTC/EFV, TDF/FTC/LPV/r | 5.37 | 190 | 2 | Intermediate | TDF/3TC/DTG/DRV/r |
| 19, F, 35 | TDF/3TC/DTG (29) | D4T/3TC/NVP, AZT/3TC/LPV/r | 6.01 | 33 | 21 | High | TDF/FTC/DRV/r |
| 20, F, 27 | TDF/3TC/DTG (22) | TDF/FTC/EFV, AZT/3TC/LPV/r, AZT/3TC/ATV/r | 5.19 | 44 | 6 | High | TDF/FTC/DRV/r |
| 21, M, 35 | TDF/3TC/DTG (29) | ART naïve | 5.07 | 250 | 13 | Intermediate | TDF/FTC/DRV/r |
| 22, F, 31 | AZT/3TC/DTG (21) | TDF/FTC/EFV | 3.01 | 178 | 20 | High | TDF/FTC/DRV/r |
| 23, F, 41 | AZT/3TC/DTG (21) | TDF/FTC/EFV | 4.05 | 360 | 15 | High | TDF/FTC/DRV/r |
| 24, M, 43 | TDF/3TC/DTG (39) | TDF/FTC/EFV, AZT/3TC/DTG | 6.23 | 78 | 32 | High | TDF/FTC/DRV/r |
| 25, F, 34 | AZT/3TC/DTG (36) | TDF/FTC/EFV | 4.72 | 366 | 31 | High | TDF/FTC/DRV/r |
| 26, F, 34 | AZT/3TC/DTG (33) | TDF/FTC/EFV | 4.06 | 131 | 24 | High | TDF/FTC/DRV/r |
| 27, F, 52 | AZT/3TC/DTG (33) | TDF/FTC/EFV | 4.92 | ND | 29 | High | TDF/FTC/DRV/r |
| 28, M, 58 | TDF/3TC/DTG (38) | TDF/FTC/EFV, AZT/3TC/DTG | 5.03 | 21 | 34 | High | TDF/FTC/DRV/r |
| 29, M, 31 | AZT/3TC/DTG/ DRV/r (24) | TDF/FTC/EFV | 3.48 | 52 | 1 | High | TDF/FTC/DRV/r |
| 30, M, 38 | ABC/3TC/DTG (26) | TDF/FTC/EFV | 3.68 | 220 | 17 | Intermediate | TDF/FTC/DRV/r |
| 31, F, 35 | AZT/3TC/DTG (23) | TDF/FTC/EFV | 4.50 | 353 | 16 | High | TDF/FTC/DRV/r |
| 32, F, 40 | AZT/3TC/DTG (24) | TDF/FTC/EFV | 3.97 | ND | 8 | High | TDF/FTC/DRV/r |
| 33, F, 50 | AZT/3TC/DTG (31) | TDF/FTC/EFV | 4.12 | 374 | 15 | Intermediate | TDF/FTC/DRV/r |
| 34, M, 34 | TDF/3TC/DTG (32) | TDF/FTC/EFV | 4.79 | 112 | 23 | Intermediate | TDF/FTC/DRV/r |
| 35, M, 35 | TDF/3TC/DTG (39) | TDF/FTC/EFV, AZT/3TC/DTG | 4.49 | 334 | 27 | High | TDF/FTC/DRV/r |
| 36, F, 33 | AZT/3TC/DTG (29) | TDF/FTC/EFV, AZT/3TC/LPV/r | 4.42 | 432 | 14 | High | TDF/FTC/DRV/r |
| 37, M, 47 | TDF/3TC/DTG (30) | TDF/FTC/EFV, ABC/3TC/LPV/r | 4.02 | 158 | 23 | High | TDF/FTC/DRV/r |
| 38, M, 39 | TDF/3TC/DTG (36) | TDF/FTC/EFV, AZT/3TC/DTG | 3.68 | 481 | 33 | Low | TDF/FTC/DRV/r |
| 39, F, 31 | TDF/3TC/DTG (24) | TDF/FTC/EFV, AZT/3TC/DTG | 4.05 | 277 | 16 | Intermediate | TDF/FTC/DRV/r |
| 40, F, 25 | TDF/3TC/DTG (31) | TDF/FTC/NVP, TDF/FTC/EFV, AZT/3TC/ATV/r, AZT/3TC/DTG | 3.58 | 338 | 14 | High | TDF/FTC/DRV/r |
| 41, M, 36 | ABC/3TC/DTG (23) | TDF/FTC/EFV, TDF/3TC/LPV/r | 4.37 | 211 | 13 | High | TDF/FTC/DRV/r |
| 42, F, 38 | TDF/3TC/DTG (40) | TDF/FTC/EFV, TDF/3TC/LPV/r, AZT/3TC/DTG | 4.32 | 291 | 35 | Intermediate | TDF/FTC/DRV/r |
| 43, F, 38 | AZT/3TC/DTG (24) | TDF/FTC/EFV, AZT/3TC/LPV/r | 3.90 | 79 | 18 | Intermediate | TDF/FTC/DRV/r |
| ^a^Age is at the time of application for genotyping.  ^b^Including single-drug changes due to side effects and listed from oldest to most recent.  ^c^The data for CD4 cell count and HIV-1 RNA were recorded at the time of virologic failure on DTG-based regimen.  ^d^From the time of the first HIV-1 RNA >1000 copies/mL on the DTG-based regimen to the application date for genotyping.  ^e^Resistance was classified with the Stanford algorithm as susceptible (score <10), potential low (10 – 14), low (15 – 29), intermediate (30 – 59), or high (≥60). | | | | | | | |
| *DTG, dolutegravir; ABC, abacavir; 3TC, lamivudine; EFV, efavirenz; TDF, tenofovir disoproxil; FTC, emtricitabine; AZT, zidovudine; D4T, stavudine; NVP, nevirapine; DRV/r, ritonavir boosted darunavir; LPV/r, ritonavir boosted lopinavir; ATV/r, ritonavir boosted atazanavir.* | | | | | | | |

**Supplementary Table S2. Summary of Genotypic Antiretroviral Resistance Testing Results**

| **Case No.** | **Current Regimen (Duration, mo)** | **INSTI Resistance Mutations** | **DTG Resistance Level^a^** | **NRTI Resistance Mutations** | **NNRTI Resistance Mutations** | **PI Resistance Mutations** |
| --- | --- | --- | --- | --- | --- | --- |
| 1 | AZT/3TC/DTG (14) | N155H, R263K | High | M184V | K103N, P225H | None |
| 2 | TDF/3TC/DTG (18) | G118R, E157Q | Intermediate | M41L, E44D, D67N, T69D, L74V, M184V, L210W,  T215Y | A98G, K103N, V108I,  H221H/Y, F227L,  M230L | V82A |
| 3 | AZT/3TC/DTG (24) | H51H/R, G118R, E138K, R263K | High | M184V | K103N, V108I, E138K | None |
| 4 | TDF/3TC/DTG (13) | R263K | Intermediate | M184V, T215Y | A98G, K103N,  V179I/V, P225H | None |
| 5 | AZT/3TC/DTG (11) | R263K/R | Intermediate | M184V | None | V82A |
| 6 | AZT/3TC/DTG (19) | Q95K, E138K,  S147G, N155H | Intermediate | D67D/N, K70K/R, M184M/V, K219E/K | K103K/N, V106M/V | None |
| 7 | AZT/3TC/DTG (60) | T66I, G118R, E138K | High | D67G, K70R, M184V,  T215F, K219E | L100I, K103N, V179I/T | None |
| 8 | AZT/3TC/DTG (24) | H51Y | Potential low | M184V | None | None |
| 9 | AZT/3TC/DTG (23) | E138A, G140A, Q148R | High | D67N, K70R, M184V, K219Q | K103N, G190A,  P225H, F227L | None |
| 10 | AZT/3TC/DTG (24) | G118R, E138A | High | M41L, D67N, M184V, T215Y | K101E, K103S, E138A, V179I/V, G190A | Q58E |
| 11 | AZT/3TC/DTG (24) | T66I, G118R, E138K, E157Q | High | M41L/M, K70R, M184V, T215F/V, K219Q | A98G, V106I/M/V, V179D, Y181C, G190A | None |
| 12 | AZT/3TC/DTG (21) | G118R, R263K | High | M184V | K103N, V108I, P225H, K238T | None |
| 13 | AZT/3TC/DTG (20) | E138K, S147G, Q148R, N155H | High | M184V | V179I/M/V, Y181F/Y | None |
| 14 | TDF/3TC/DTG (11) | E157Q, R263K | Intermediate | A62V, K65R, Y115F/Y, M184V | None | None |
| 15 | TDF/3TC/DTG (23) | T66A, G118R, E138K, G163R | High | M41L, D67N, K70N,  M184V,  T215Y | K103N, P225H | None |
| 16 | TDF/3TC/DTG (28) | R263K | Intermediate | L74I, M184V | K103N, P225H | None |
| 17 | TDF/3TC/DTG (23) | G118R | Intermediate | K70R, K219Q | K101E, E138A, G190A | None |
| 18 | TDF/3TC/DTG (6) | R263K | Intermediate | K70H/K/N/Q, L74I, Y115F, M184V | K101R, K103N, V108I,  Y181C, H221Y, K238N | None |
| 19 | TDF/3TC/DTG (29) | E138K, G140A, Q148K, S230R | High | No amplification | No amplification | No amplification |
| 20 | TDF/3TC/DTG (22) | E138K, G140A, Q148K | High | M41L/M, M184V, T215Y | None | None |
| 21 | TDF/3TC/DTG (29) | A49G, G163R, R263K | Intermediate | D67N, K70E, M184V | V179D | None |
| 22 | AZT/3TC/DTG (21) | G118R, E138K, E157Q | High | M184V | K103N | None |
| 23 | AZT/3TC/DTG (21) | T66I, G118R, E138A/E/K/T | High | D67N, K70R, M184V, K219E | A98G, K103N, V179I/V, P225H | None |
| 24 | TDF/3TC/DTG (39) | G118R, E138E/K, E157Q | High | M41L, D67N, K70R, V75M, M184V, T215Y, K219K/Q | K101E, V106I, G190A | None |
| 25 | AZT/3TC/DTG (36) | L74L/M, G118R, E138K, R263K | High | M184V | K103N, E138A, K238T | None |
| 26 | AZT/3TC/DTG (33) | G118R, E138K | High | M184V, K219K/R | V106I | None |
| 27 | AZT/3TC/DTG (33) | G118R, E138K, R263K | High | M184V | K103N, V108V/I, P225H | None |
| 28 | TDF/3TC/DTG (38) | A128T, E138K, G140A, Q148K | High | K70E, M184V | K103N | None |
| 29 | AZT/3TC/DTG/ DRV/r (24) | G118R, E138K, E157Q | High | L74I, Y115F, M184V, T215Y | K103S, V106M, E138G | None |
| 30 | ABC/3TC/DTG (26) | E157Q, R263K | Intermediate | K65R, K70T, L74I, Y115F, M184V | K103S, V106I, E138G | L33F/L |
| 31 | AZT/3TC/DTG (23) | T66A, G118R,  E138K, E157Q | High | M184V | Y188L | None |
| 32 | AZT/3TC/DTG (24) | E138K, , S147G, N155H, R263K | High | K65R, D67G, S68G, M184V | V106M, E138A, V179D, M230L | None |
| 33 | AZT/3TC/DTG (31) | R263K | Intermediate | S68S/G, M184V | A98G, K103N, E138Q | None |
| 34 | TDF/3TC/DTG (32) | E157Q, R263K | Intermediate | K70Q, Y115F, M184V | None | L10L/F |
| 35 | TDF/3TC/DTG (39) | E138K, G140A, S147G, Q148R | High | D67N, K70R, M184V,  T215F, K219E | K101E, Y181C, G190A | None |
| 36 | AZT/3TC/DTG (29) | T66A, L74I/M,  G118R, E138K, G149G/A | High | M184V | K103N, P225H | None |
| 37 | TDF/3TC/DTG (30) | T66I, L74M,  G118R, E138K | High | M41L, S68G, M184V,  T215F/Y | A98G, V106I, Y188L | L10F, I54V, Q58E, L76V, V82A |
| 38 | TDF/3TC/DTG (36) | Q95Q/K, T97T/A, E138E/K, N155H | Low | D67N, K70R, M184V, K219E | K103N, P225H | None |
| 39 | TDF/3TC/DTG (24) | R263K | Intermediate | K70E, M184V | None | None |
| 40 | TDF/3TC/DTG (31) | T66I, G118R,  E138K, G149G/A, | High | S68G, M184V | K101H, Y181C, G190A, H221Y | None |
| 41 | ABC/3TC/DTG (23) | E138A, G140A,  Q148R | High | M184V | None | None |
| 42 | TDF/3TC/DTG (40) | T97A, S147G, N155H, E157Q | Intermediate | K65K/R, K70G/R, M184V | V106M, V179D, F227L | None |
| 43 | AZT/3TC/DTG (24) | H51Y, R263K | Intermediate | M184V | K103N | None |
| ^a^Resistance was classified with the Stanford algorithm as susceptible (score <10), potential low (10 – 14), low (15 – 29), intermediate (30 – 59), or high (≥60). | | | | | | |
| *DTG, dolutegravir; ABC, abacavir; 3TC, lamivudine; TDF, tenofovir disoproxil; AZT, zidovudine; DRV/r, ritonavir boosted darunavir; INSTI, integrase strand transfer inhibitor; NRTI, nucleoside reverse transcriptase inhibitor; NNRTI, non-nucleoside reverse transcriptase inhibitor; PI, protease inhibitor.* | | | | | | |
